# Early Prediction of Cardiomyopathy in Pediatric Cancer Survivors Using Echocardiographic Video‐Based Deep Learning

**DOI:** 10.1111/echo.70643

**Published:** 2026-10-22

**Authors:** Surbhi Sharma, Brandy Martinez Di Pietro, Ananya Nandula, Tanishka Goplani, Hanpeng Chen, Saro Armenian, Aarti Bhat, Nancy Blythe, William Border, Kasey J. Leger, Wendy M. Leisenring, Lillian R. Meacham, Shanti Narasimhan, Paul C. Nathan, Ritu Sachdeva, Karim Sadak, Kayla Stratton, Eric J. Chow, Lindsay A. Edwards, Patrick M. Boyle

**Affiliations:** ^1^ Department of Bioengineering University of Washington Seattle Washington USA; ^2^ Seattle Children's Hospital Seattle Washington USA; ^3^ Departments of Pediatrics and Population Sciences City of Hope Duarte California USA; ^4^ Department Of Pediatrics University of Washington Seattle Washington USA; ^5^ Clinical Research and Public Health Sciences Divisions Fred Hutchinson Cancer Center Seattle Washington USA; ^6^ Department Of Pediatrics Emory University Atlanta Georgia USA; ^7^ Aflac Cancer and Blood Disorders Center of Children's Healthcare of Atlanta and Department of Pediatrics Emory University Atlanta Georgia USA; ^8^ Department Of Pediatrics University of Minnesota, Masonic Children's Hospital Minneapolis Minnesota USA; ^9^ Department Of Pediatrics University of Toronto, The Hospital for Sick Children Toronto Ontario Canada; ^10^ Departments Of Pediatrics Duke University Medical Center Durham North Carolina USA; ^11^ Division of Cardiology University of Washington Seattle Washington USA; ^12^ Institute For Stem Cell and Regenerative Medicine University of Washington Seattle Washington USA; ^13^ Center For Cardiovascular Biology University of Washington Seattle Washington USA

**Keywords:** cancer survivors, cardiomyopathies, cardiotoxicity, echocardiography, machine learning

## Abstract

**Purpose:**

Cardiomyopathy (CM) is a leading cause of late mortality in childhood cancer survivors, but it is often identified only after irreversible cardiac remodeling has already occurred. In this proof‐of‐concept study, we assessed whether deep learning applied to routine echocardiographic videos can identify features predictive of future CM.

**Methods:**

We analyzed parasternal short‐axis clips from cancer survivors ≤21 years old at cancer diagnosis. 3D deep convolutional neural networks were trained to classify CM‐positive versus CM‐negative studies from echocardiograms conducted up to five years prior to CM diagnosis. The performance was assessed on internal hold‐out validation (Center 1) and external validation cohorts (Centers 2, 3). The model's prognostic value was evaluated by stratifying patients into high‐ and low‐risk groups for developing CM, and probability of future CM was assessed using cumulative incidence and proportional hazards regression analysis.

**Results:**

The model achieved an area under the receiver operating characteristic curve (AUROC) of 0.87±0.05 and an area under the precision–recall curve (AUPRC) of 0.74±0.07 on the internal hold‐out set. External validation showed near‐chance single‐study discrimination (AUROC 0.55±0.02 and 0.60±0.03; AUPRC 0.22±0.02 and 0.21±0.03 for Centers 2 and 3, respectively). Nevertheless, analysis of the external cohort showed significantly higher probability of CM in high‐ versus low‐risk groups (hazard ratio 2.78, 95% CI = 1.52–5.10, *p* = 0.00056).

**Conclusion:**

Deep learning models have the potential to identify subtle echocardiographic markers years before CM diagnosis in pediatric cancer survivors and could serve as a preliminary step toward risk‐guided CM surveillance. Further refinement and validation are needed to determine the utility of deep learning for early identification of pathologic cardiac remodeling in this population.

## Graphical abstract

Deep learning pipeline for cardiomyopathy prediction in pediatric cancer survivors using echocardiographic videos. Childhood cancer survivors face increased cardiomyopathy risk following cardiotoxic cancer therapy. We tested whether three‐dimensional deep learning could identify early cardiac changes in routine surveillance echocardiographic videos. The model detected subtle imaging features associated with future cardiomyopathy before clinical diagnosis. Although external validation showed reduced performance, time‐to‐event analysis demonstrated prognostic value. These proof‐of‐concept findings support the potential for artificial intelligence to complement established risk factors and enable earlier, personalized surveillance, while emphasizing the need for further refinement and validation before clinical implementation.

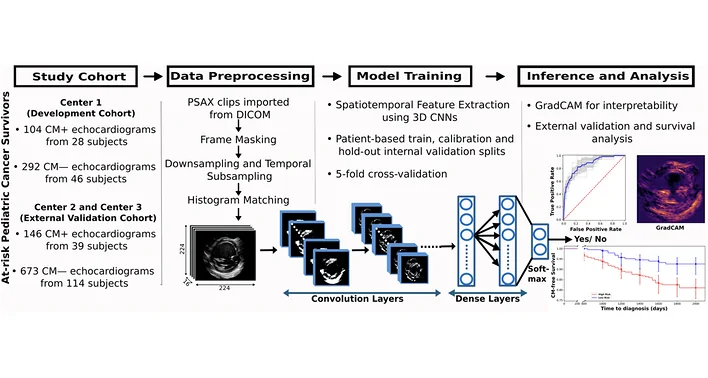

**Abbreviations**
AUPRC: area under the precision–recall curve
AUROC: area under the receiver operating characteristic curve
CM: cardiomyopathy
DCNN: deep convolutional neural network
DL: deep learning
Grad‐CAM: Gradient‐weighted Class Activation Mapping
HR: hazard ratio
LV: left ventricular
NPV: negative predictive value
PPV: positive predictive value
PSAX: parasternal short‐axis view
REDCap: Research Electronic Data Capture
ReLU: rectified linear unit

## 1 Introduction

Pediatric cancer survival rates continue to improve, with a 5‐year survival rate of approximately 85% and nearly 500,000 survivors currently estimated to be living in the United States [1, 2]. The vast majority of these survivors, however, will develop a chronic health condition by age 40 years [3]. As such, there is an urgent need to better understand and address the long‐term morbidity and mortality linked to the cancer treatments that have improved survival rates. Cardiovascular disease, particularly treatment‐related cardiomyopathy (CM), is a leading cause of premature morbidity and mortality among pediatric cancer survivors [4, 5]. Current guidelines recommend routine echocardiographic screening for high‐risk patients to detect early signs of CM such as left ventricular (LV) systolic dysfunction and abnormal ventricular geometry [6]. Echocardiographic assessments, however, often are affected by intra‐ and inter‐observer variability, limiting the sensitivity and raising concerns about the prognostic value of these assessments [7, 8]. Observable changes typically suggest that irreversible cardiac remodeling has already occurred [9]. Hence, methods that highlight subtle mechanisms of early disease progression and enhance earlier detection of CM in this population could have a major clinical effect.

Deep learning (DL) techniques excel at processing complex patterns within large datasets (e.g., medical images). By learning important features from the medical images, DL can track disease progression and predict outcomes, enabling timely and targeted interventions [10]. Deep convolutional neural networks (DCNNs), a form of DL, have shown great promise in various medical fields, including cardiology [11, 12, 13], improving disease diagnosis, prognosis, and clinical decision‐making. However, DL applications are still emerging in the field of cardio‐oncology and are particularly scarce in pediatric settings [14, 15, 16].

Despite advances in cardiac modeling, no method exists to capture the temporal dynamics of CM‐associated cardiac remodeling in pediatric patients. This gap exists due to several challenges: [13, 17] lack of publicly available pediatric echocardiogram datasets; the labor‐intensive process of data labeling and preprocessing; and the rarity of pediatric cardiovascular diseases, which can lead to dataset imbalance (i.e., very few affected individuals relative to those unaffected). As a result, DL has not yet fully realized its potential in pediatric cardio‐oncology. Addressing these issues, including variability in patient demographics and imaging technologies, is crucial to unlocking the full potential of DL for this at‐risk population.

To address these challenges, we developed DL models to assess the feasibility of DL‐based approach for detecting early imaging features associated with future CM development using echocardiographic videos from a large, multi‐institutional cohort of pediatric cancer survivors (Graphical Abstract). Leveraging 3D DCNNs trained on parasternal short‐axis (PSAX) echocardiographic clips, our approach captures both spatial and temporal dynamics of cardiac motion. We hypothesize that these models could identify subtle imaging signatures predictive of future CM—even years before clinical diagnosis. By evaluating model performance across multiple periods preceding CM onset, our study aims to demonstrate the feasibility of DL‐based risk prediction in pediatric cardio‐oncology and lay the groundwork for earlier, precision‐guided surveillance strategies.

## 2 Patients and Methods

### 2.1 Study Population

We used a retrospective dataset comprising subjects diagnosed with cancer before age 21 years and treated and followed at one of three participating centers (all large children's hospitals; referred to as Center 1, Center 2, Center 3) for model development and validation. All subjects were childhood cancer survivors at risk for CM due to exposure to cardiotoxic cancer therapies (i.e., anthracyclines and/or chest radiation) who had survived at least 1 year after completing initial treatment. As part of routine surveillance, these survivors are monitored with echocardiograms during treatment (typically prior to anthracycline‐containing treatment courses); at end of cancer‐therapy; and at regular intervals during survivorship, typically varying from annually to every 5 years based on national pediatric oncology long‐term follow‐up guidelines in place during the study period [18]. Given the retrospective nature of this study, all eligible subjects meeting inclusion criteria were included, and no a priori sample size calculation was performed.

Data from Center 1 were used for model development and internal validation, while data from the other two centers were reserved for external validation. As previously published, we defined CM‐positive (CM+) cases as at‐risk survivors showing reduced systolic function (shortening fraction ≤28% or ejection fraction ≤50%) on two or more routine 2D echocardiograms, with at least one study performed post‐cancer therapy, or in cases where heart failure treatment lasting at least six months, began after a single echocardiogram [19]. CM negative (CM−) subjects (our control population) were at‐risk survivors (exposed to anthracyclines and/or chest radiotherapy) who underwent regular echocardiogram monitoring without developing CM after completing therapy [19]. Specifically, CM− subjects were required to have a shortening fraction of ≥30% and an ejection fraction of ≥55%, with no echocardiographic reports indicating qualitative findings suggestive of CM. Exclusion criteria for both CM+ and CM− subjects included the presence of congenital heart disease or genetic syndromes known to affect cardiac function (e.g., trisomy 21).

For all eligible subjects, trained research personnel at each center extracted relevant demographic and clinical information from medical records and entered the data into a centralized Research Electronic Data Capture (REDCap) database [20]. Deidentified echocardiogram studies, labeled only with a study‐specific identifier, were uploaded in DICOM format to PICOM365 (SciImage, Los Altos, CA), which is a secure, cloud‐based imaging repository. Institutional review boards at all three centers approved the study protocol with a waiver of informed consent.

### 2.2 Ethics in Research

This study was reviewed and approved by the Fred Hutchinson Cancer Center Institutional Review Board (IR file 8432) and the NIH Central Institutional Review Board (protocol number ALTE11C2). Both committees granted a waiver of informed consent because the research involved analysis of existing medical records.

### 2.3 Echocardiographic Data Selection and Processing

DICOM‐format videos were retrieved from a secure, cloud‐based server, and each study was reviewed to ensure appropriate view selection. PSAX Echocardiographic video clips at the level of papillary muscles were manually identified by trained sonographers or study physicians. This view was chosen in accordance with pediatric imaging guidelines, because it provides optimal visualization of LV size, wall thickness, and function [7]. Video clips were accepted in grayscale or other standard display formats (e.g., sepia). Studies with suboptimal image quality due to factors such as lung artifact or limited acoustic windows were retained to preserve the real‐world variability present in clinical imaging. Next, one PSAX video clip per echocardiogram was randomly selected. To protect patient confidentiality and ensure consistency in input data, each frame was masked to remove identifying information, overlaid annotations, and embedded interface text. To reduce variability due to acquisition protocols and align with common DL practices [21], all videos were standardized to 32 frames per second. Next, temporal subsampling was performed by selecting every alternate frame from the first 32 frames to create a 16‐frame clip, approximating a single cardiac cycle. This approach preserved essential cardiac motion dynamics while minimizing redundancy and ensuring consistent input length across studies. Video frames were downsampled spatially to 224 × 224 pixels. To address variability in brightness and contrast across studies, histogram matching [22] was applied, enhancing the visual consistency of input frames without altering underlying structural features. We evaluated data augmentation (random rotation, translation, blur, gamma correction, additive noise, and brightness adjustment during training) as an additional strategy for improving robustness to inter‐site appearance differences. This did not improve cross‐validation performance relative to histogram matching alone and was not retained in the final pipeline. All processed videos were converted to grayscale and used as inputs to 3D DCNN models.

### 2.4 Data Labeling

To investigate echocardiographic patterns preceding the onset of CM, we curated datasets by resampling the CM+ data according to defined time intervals relative to CM diagnosis. Specifically, three periods based on CM+ studies were established: 1) CM_all_ (included prediagnosis studies up to 5 years before CM diagnosis), 2) CM_1–5_ (prediagnosis studies within 1 to 5 years of CM diagnosis; excluding those within 1 year of CM), and 3) CM_1–3_ (only prediagnosis studies within 1 to 3 years of CM diagnosis). The year preceding diagnosis was excluded from models CM_1–5_ and CM_1–3_ to address any potential diagnostic gray zone with imperfect echocardiographic discrimination between CM+ and CM− subjects. Studies from 4 to 5 years prediagnosis were excluded from CM_1–3_, as echocardiographic differences can be absent or inadequately discernible several years before diagnosis. For subjects who developed CM, all available PSAX echocardiographic studies falling within the defined prediagnosis intervals were included. All available CM− echocardiograms were included across models. More than one video per subject could be included in a given model when multiple eligible studies were available.

Separate models were trained for each period to evaluate if predictive performance varied by temporal distance from CM diagnosis. This approach enabled assessment of model sensitivity to early changes in cardiac structure and function, including in otherwise asymptomatic subjects—and supported our broader objective of identifying echocardiographic markers of cardiac remodeling in at‐risk survivors.

### 2.5 DCNN Architecture and Performance

Three separate DCNNs with the same model architecture were trained for the three established periods; each DCNN was tasked with classifying echocardiographic video clips as CM+ or CM−. DCNNs were implemented using the Keras (version 2.15) API in Python (version 3.9) with a TensorFlow (version 2.15) backend [23]. The model consisted of a 3D convolutional neural network designed to extract spatiotemporal features from grayscale video clips consisting of 16 frames (input dimensions:16 × 224 × 224). The model architecture comprised six 3D convolutional layers, each followed by batch normalization and rectified linear unit (ReLU) activation to promote training stability and introduce nonlinearity (Figure S1). Selected layers also included maximum pooling operations to progressively reduce spatial dimensions and enable hierarchical feature extraction. To mitigate overfitting and improve generalization, an L2 kernel regularizer was applied to the convolutional layers, penalizing large weights during training.

After the final convolutional block, a global average pooling layer aggregated spatiotemporal features across the entire video clip. The resulting feature vector was passed to a fully connected dense layer with 256 units, followed by ReLU activation and a dropout layer for regularization. The output layer was a dense layer with Softmax activation to generate class probabilities for CM+ versus CM− subjects. The model was compiled using Adam optimizer and categorical cross‐entropy loss function [24]. Hyperparameters—including batch size, epochs, learning rate, and dropout rate—were tuned based on internal validation performance. Final hyperparameter settings are reported in Table S1.

A training, calibration, and internal hold‐out validation split of 60:20:20 was used for the development cohort. In standard machine learning terminology, the calibration set often is referred to as the validation set, and the hold‐out validation set as the test set [25]. To enhance clarity and avoid confusion, however, we adopted the terms “calibration” and “internal hold‐out validation” in this work. Additionally, 5‐fold cross‐validation was performed between the training and calibration sets to optimize model performance. Subsequently, the performance of the internal hold‐out validation set was evaluated for every fold. To prevent data leakage, train‐calibration‐validation splits were performed based on patients rather than echocardiographic studies. This patient‐based split ensures that all the data from a given patient is contained within a single split (either train, calibration, or validation), thereby reducing the risk of information leakage and improving the model's generalizability. Further, no patient contributed studies to more than one model's (CM_all_, CM_1–5_, CM_1–3_) validation set to avoid subject‐level correlation. Receiver operating and precision‐recall curves were generated for each of the five folds. Corresponding area under the curve metrics, area under the receiver operator characteristic curve (AUROC) and area under the precision‐recall curve (AUPRC) were calculated for each fold and averaged to generate mean and standard deviation values. AUPRC is considered highly informative for assessment of imbalanced datasets [26]. Several additional standard metrics were used to evaluate DCNN performance including sensitivity, specificity, positive/negative predictive values (PPV/NPV), F1‐score, and accuracy. To further evaluate generalizability, external validation was conducted using studies from Center 2 and Center 3. The CM_all_ model was carried forward for the external holdout analysis because it was the best‐performing model in the development cohort. Probability calibration was assessed in the internal hold‐out cohort and external validation cohorts by plotting observed versus predicted CM risk.

The extent of our study's compliance with contemporary reporting recommendations for prediction models using machine learning is documented in the completed TRIPOD+AI checklist provided as supplementary material.

### 2.6 Model Interpretability

To enhance model interpretability and evaluate the spatial regions influencing CM predictions, we applied Gradient‐weighted Class Activation Mapping (Grad‐CAM) [27] to the final convolutional block of the trained 3D DCNN model. Grad‐CAM is a visualization technique that highlights salient regions in the input data relevant to a model's decision. It was used to generate class‐discriminative localization maps, highlighting the regions of each echocardiogram frame that most contributed to the model's prediction of CM+ versus CM−. For each selected study, Grad‐CAM heatmaps were computed and overlaid on the corresponding input frames to enable qualitative assessment of model attention and ensure that the model focuses on clinically relevant cardiac regions.

### 2.7 Time‐to‐Event Analysis

To assess the prognostic utility of the model's predictions, we conducted time‐to‐event analysis using external validation studies from Center 2 and Center 3. For CM+ subjects, we included studies that were acquired at least 2 years prior to CM diagnosis. Given that CM− subjects may develop cardiomyopathy in the future, we excluded the final echocardiogram and any echocardiograms performed within the preceding year for CM− subjects. For each study, a prediction probability of future CM was obtained using the CM_all_ model. Echocardiograms were stratified into high‐risk and low‐risk groups based on a predefined threshold derived from Youden index [25]. This threshold was determined using data from Center 1 only, and no information from echocardiograms from Centers 2 and 3 were used for model calibration. Confidence intervals using the Wilson score method for sensitivity and specificity at this threshold, computed on the ensembled predictions across all five cross‐validation folds, are reported in Table S2. As an exploratory analysis, we also computed site‐specific operating thresholds derived independently within Centers 2 and 3 (Table S3). However, we retained the Center 1‐derived threshold as our primary analysis because a threshold derived from a site's own outcomes could not be applied prospectively at that site and evaluating performance at a threshold chosen from the same labels being assessed constitutes a form of data leakage. Next, cumulative incidence curves were constructed to estimate time‐to‐event (CM diagnosis) distributions for the two risk groups. A log‐rank test was performed to assess if the time to CM diagnosis differed significantly between the high‐risk and low‐risk groups. Based on Cox proportional hazards model, a hazard ratio (HR) was calculated to quantify the relative risk of CM diagnosis between the high versus low‐risk echo groups. Proportionality assumptions were confirmed using Schoenfeld residuals. There was no competing mortality in this analysis. Discrimination was assessed using Harrell's C‐index (concordance index).

## 3 Results

The development cohort included PSAX video clips extracted from 396 unique longitudinal echocardiograms from 74 at‐risk childhood cancer survivors treated at Center 1. The echocardiograms were performed between 2007 and 2023. Of the 396 studies, 104 were from 28 subjects with newly diagnosed or future CM (CM+) and 292 were from 46 subjects without CM during follow‐up (CM−). All CM+ subjects in the development cohort met CM criteria based on reduced LV systolic function on two or more echocardiograms. All 104 studies from CM+ subjects were obtained within 5 years prior to CM diagnosis. Of these, 70 were obtained at least 1 year before CM diagnosis and 40 were obtained within the 1‐ to 3‐year prediagnosis window. The external validation cohort included PSAX video clips extracted from 819 unique longitudinal echocardiogram studies from 153 at‐risk subjects treated either at Center 2 or Center 3. The echocardiograms were performed between 2004 and 2023. In the external validation cohort, 36 CM+ subjects met CM criteria based on reduced LV systolic function on two or more echocardiograms; 3 met criteria based on a reduced LV systolic function on a single echocardiogram with documentation of heart failure medication treatment. Of the 819 studies, 146 were from 39 CM+ subjects with newly diagnosed or future CM and 673 were from 114 CM− subjects. Median age at cancer diagnosis was 6 (Q1–Q3: 3.0–11.0) years with median 8.7 (Q1–Q3: 6.5–11.7) years of follow‐up since cancer diagnosis. For CM+ subjects, median time from cancer diagnosis to cardiomyopathy diagnosis was 5.6 (Q1–Q3: 1.2–10.1) years. Other demographic and treatment characteristics of CM+ and CM− subjects are shown in Table 1.

**TABLE 1:** Demographic and clinical characteristics of the development (Center 1) and external validation (Centers 2 and 3) cohorts.

Characteristic^a^	Development cohort		External validation cohort	
	CM + N = 28	CM − N = 46	CM + N = 39	CM − N = 114
Female sex	10 (36)	24 (52)	16 (41)	51 (45)
Race/ethnicity^b^				
Asian/Pacific islander	1 (4)	2 (4)	9 (23)	6 (5)
Black	11 (39)	15 (33)	3 (8)	2 (2)
Hispanic	2 (7)	1 (2)	4 (10)	14 (12)
Multiracial	0 (0)	1 (2)	3 (8)	5 (4)
White	14 (50)	26 (57)	14 (36)	44 (39)
Unknown	0 (0)	1 (2)	6 (15)	43 (38)
Year of cancer diagnosis				
1994–1999	0 (0)	0 (0)	5 (13)	3 (3)
2000–2005	5 (18)	1 (2)	11 (28)	33 (29)
2006–2009	11 (39)	12 (26)	8 (21)	46 (40)
2010–2021	12 (43)	33 (72)	15 (38)	32 (28)
Age at cancer diagnosis				
0–4	10 (36)	24 (52)	13 (33)	43 (38)
5–9	6 (21)	10 (22)	15 (38)	35 (31)
10–14	8 (29)	8 (17)	7 (18)	29 (25)
15–20	4 (14)	4 (9)	4 (10)	7 (6)
Cancer type				
Leukemia	9 (32)	12 (26)	14 (36)	51 (45)
Lymphoma	4 (14)	7 (15)	7 (18)	23 (20)
Sarcoma	11 (39)	9 (20)	9 (23)	23 (20)
Other solid tumors	4 (14)	18 (39)	9 (23)	17 (15)
Anthracycline dose,^c^ mg/m^2^	374 (180–480)	188 (125–375)	250 (200–275)	191 (120–300)
Chest radiotherapy	3 (11)	3 (7)	4 (10)	17 (15)
Age at last follow‐up^d^	13.5 (12–17)	11 (8–17)	15 (12–16)	17 (14–18)
Year of echocardiogram	2013 (2011–2016)	2014 (2012–2016)	2015 (2014–2016)	2013 (2010–2015)

Abbreviation: CM, cardiomyopathy.

^a^ Values are n (%) or median (Q1–Q3).

^b^ All categories are non‐Hispanic unless otherwise noted. Center 3's institutional policies precluded systematic recording of race/ethnicity, and this accounted for 46 of 49 patients with unknown race/ethnicity among the external validation cohort.

^c^ All subjects were exposed to anthracyclines; cumulative dose based on doxorubicin equivalence.

^d^ Age at last follow‐up for CM− patients and age at CM diagnosis for CM+ patients.

Table 2 shows model performance metrics of the best DCNN models with the internal hold‐out validation set (i.e., Center 1) for the three periods we focused on. Receiver operating and precision‐recall curves for the DCNNs corresponding to the three periods are presented in Figure 1. CM_all_ model achieved the best performance, with mean AUROC = 0.87 ± 0.05, mean AUPRC = 0.74 ± 0.07, F1‐score = 0.63 ± 0.08, sensitivity = 0.75 ± 0.14, specificity = 0.79 ± 0.15, PPV = 0.58 ± 0.20, NPV = 0.91 ± 0.04, and accuracy = 0.78 ± 0.09. The performance declined for the CM_1–5_ and CM_1–3_ models, with AUROCs of 0.82 ± 0.06 and 0.77 ± 0.06, respectively, and AUPRCs of 0.34 ± 0.20 and 0.52 ± 0.15, respectively.

**TABLE 2:** DCNN model performance for the internal hold‐out validation sets from Center 1.

Metrics	CM_all_ model	CM_1–5_ model	CM_1–3_ model
**AUROC**	0.87 ± 0.05	0.82 ± 0.06	0.77 ± 0.06
**AUPRC**	0.74 ± 0.07	0.34 ± 0.20	0.52 ± 0.15
**F1‐score**	0.63 ± 0.08	0.27 ± 0.11	0.37 ± 0.19
**Sensitivity**	0.75 ± 0.14	0.52 ± 0.38	0.63 ± 0.37
**Specificity**	0.79 ± 0.15	0.82 ± 0.17	0.65 ± 0.16
**PPV**	0.58 ± 0.20	0.20 ± 0.06	0.34 ± 0.15
**NPV**	0.91 ± 0.04	0.96 ± 0.04	0.93 ± 0.07
**Accuracy**	0.78 ± 0.09	0.79 ± 0.13	0.66 ± 0.18

Values are mean ± SD across the five cross‐validation fold models, each applied without retraining to the internal hold‐out validation set.

**FIGURE 1:**
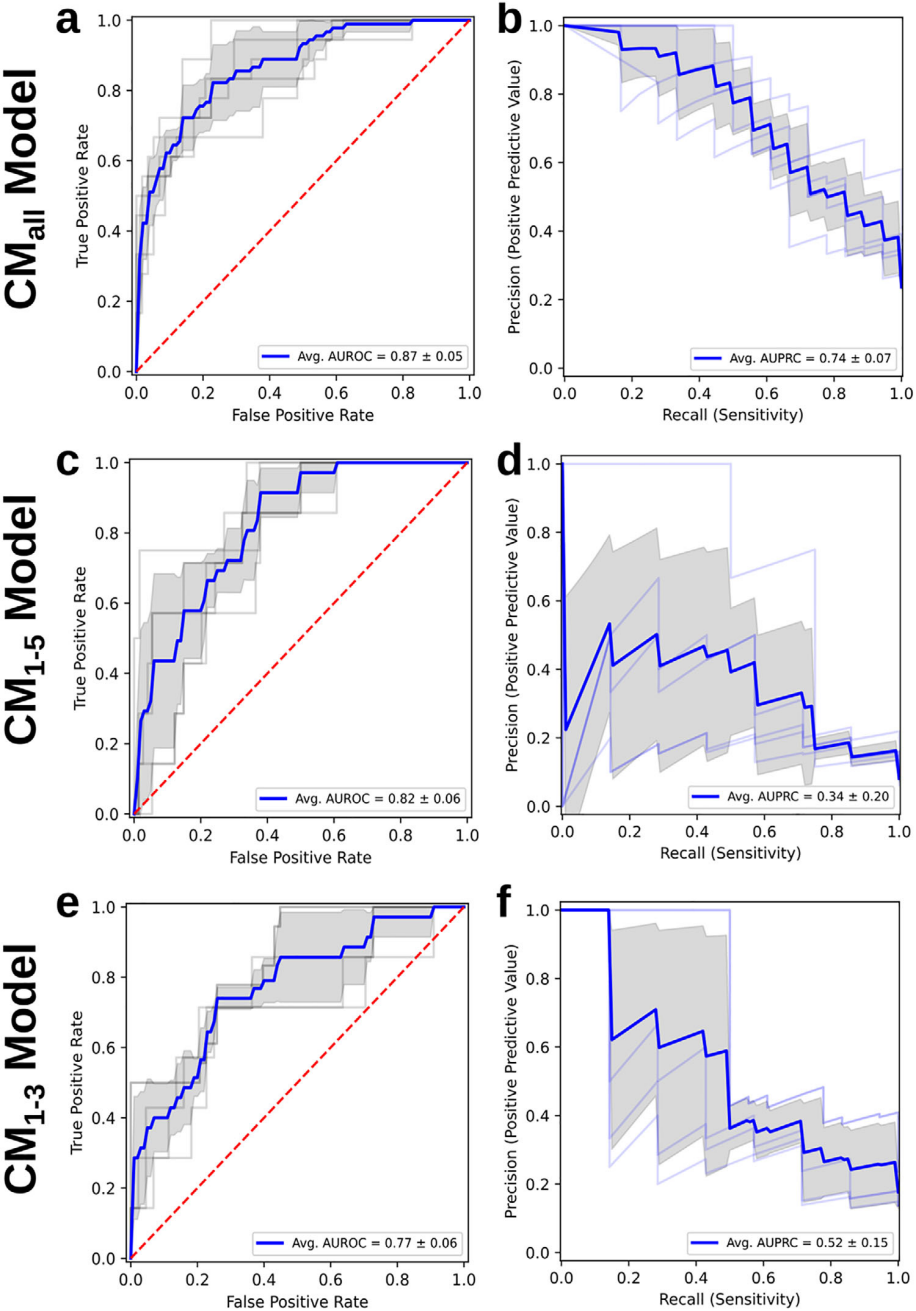
DCNN model performance on the internal hold‐out validation set. (a–b) Average receiver operating characteristic (ROC) curve and (c–d) Average precision‐recall (PR) curve for the three periods. Solid lines represent the mean performance across 5‐folds, while the grey shaded regions indicate the standard deviation across folds.

External validation was performed using the CM_all_ model across the two independent cohorts from Center 2 and Center 3. Performance metrics across these two centers are shown in Table 3. While the external AUROC (Center 2: 0.55 ± 0.02, Center 3: 0.60 ± 0.03) and AUPRC (Center 2: 0.22 ± 0.02, Center 3: 0.21 ± 0.03) were lower than internal results based on Center 1, the model retained moderate sensitivity, specificity, and NPV. Data from both external centers were combined for a time‐to‐CM analysis (Figure 2). Time‐to‐event analysis revealed a significant difference in time to CM diagnosis between the high‐risk and low‐risk groups (log‐rank *p* = 0.00056, HR = 2.78, 95% CI = 1.52–5.10), with high‐risk subjects demonstrating a significantly higher probability of future CM. The model demonstrated modest discrimination (Harrell's C‐index 0.61, 95% CI 0.55–0.68).

**TABLE 3:** CM_all_ model performance for external validation data.

Metrics	Center 2	Center 3
**AUROC**	0.55 ± 0.02	0.60 ± 0.03
**AUPRC**	0.22 ± 0.02	0.21 ± 0.03
**F1‐score**	0.34 ± 0.01	0.32 ± 0.01
**Sensitivity**	0.62 ± 0.12	0.63 ± 0.18
**Specificity**	0.52 ± 0.14	0.57 ± 0.19
**PPV**	0.24 ± 0.02	0.23 ± 0.05
**NPV**	0.85 ± 0.01	0.90 ± 0.02
**Accuracy**	0.54 ± 0.09	0.58 ± 0.13

**FIGURE 2:**
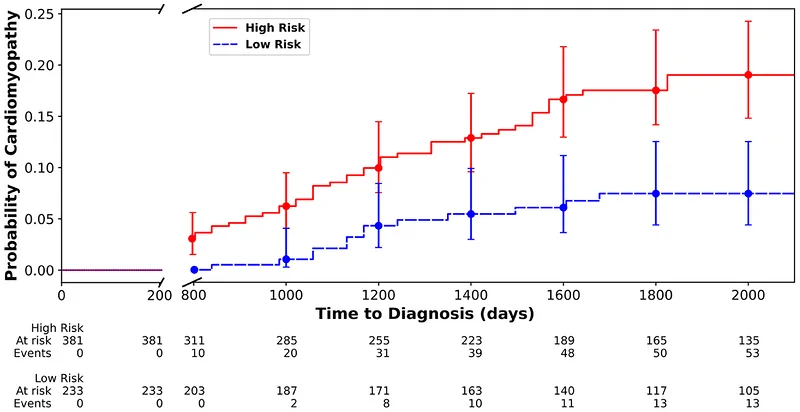
Probability of CM over time in the external validation cohort. Subjects were divided into high‐ and low‐risk groups based on predicted probability of future cardiomyopathy from the CM_all_ model. Error bars indicate 95% confidence intervals. The high‐risk group showed a significantly higher probability of developing CM (HR = 2.78, 95% CI = 1.52–5.10, log‐rank *p* = 0.00056), supporting the prognostic utility of model predictions.

Probability calibration plots are shown in Figure S2. Calibration in the internal hold‐out cohort was generally consistent with predicted risk, although variability was observed at higher predicted probabilities. In the external validation cohorts, predicted probabilities tended to exceed observed event rates, indicating overestimation of absolute CM risk.

To qualitatively assess model interpretability, the Grad‐CAM technique was applied to highlight regions of interest in the echocardiogram frames that influenced prediction outcomes. Figure 3 shows Grad‐CAM heatmaps for one CM+ and one CM− study, each rescaled to display four representative frames. Full‐frame Grad‐CAM visualizations (all 16 frames per study) for both examples are provided in Figure S3. In both cases, the model's attention was primarily focused on different layers of the heart wall and papillary muscles.

**FIGURE 3:**
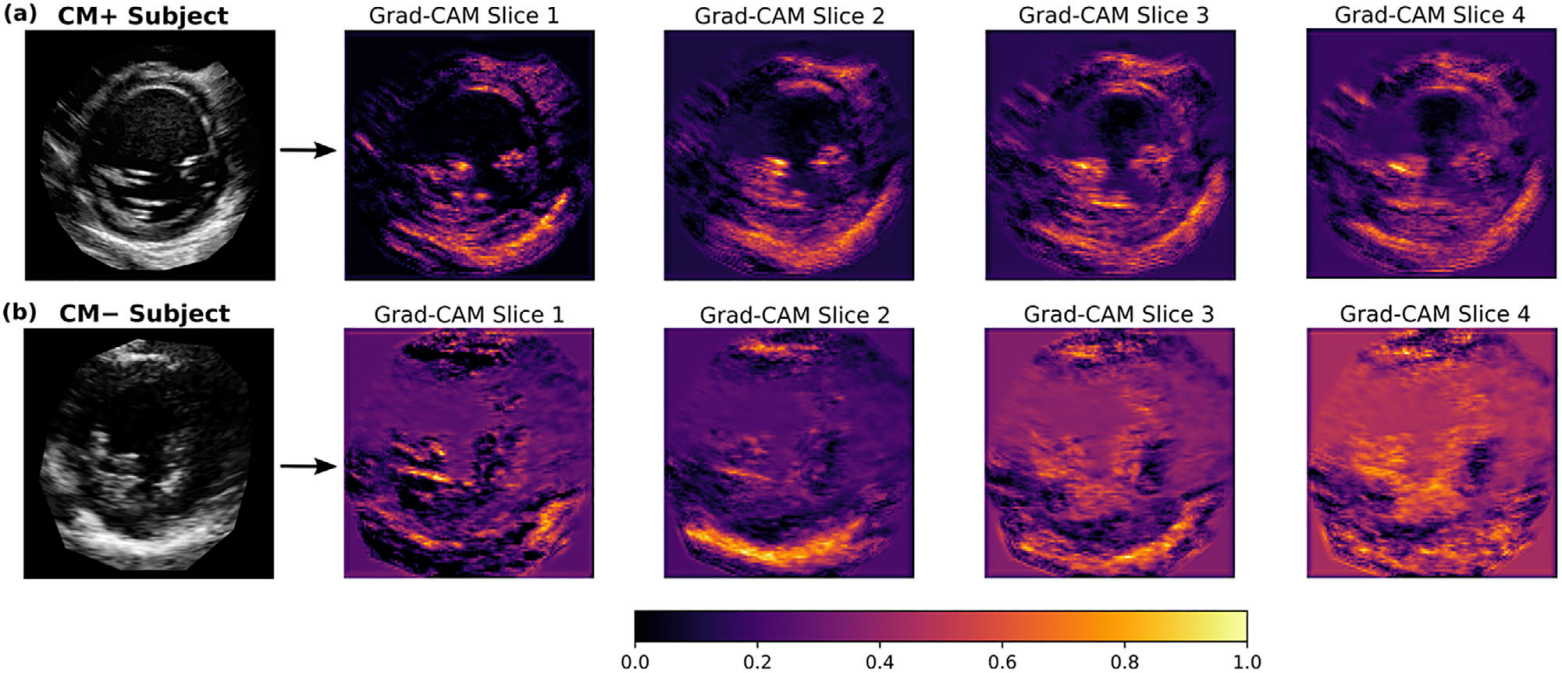
Grad‐CAM visualizations. Four representative frames (rescaled from the original 16‐frame input) for representative CM+ (a) and CM− (b) studies, illustrating the regions that most influenced model's predictions. Brighter regions represent features with greater influence on the model's prediction.

## 4 Discussion

In this study, we developed and validated DL models that leverage echocardiographic video data to detect features of cardiac remodeling in childhood cancer survivors years before CM diagnosis. The principal contribution of this study is the application of 3D spatiotemporal video modeling to predict future CM in childhood cancer survivors. This differs from prior studies (e.g., DCNN analysis of static four‐frame montages, video‐based deep learning to estimate left ventricular ejection fraction rather than future CM risk). The CM_all_ model achieved good discrimination on the internal hold‐out validation set, with a mean AUROC of 0.87 ± 0.05 and AUPRC of 0.74 ± 0.07. The model performance was more limited when subjected to external validation. Time‐to‐event analysis of data from the pooled external cohorts, however, revealed a significant difference in probability of developing CM between model‐defined high‐risk and low‐risk groups (log‐rank *p* = 0.00056, HR = 2.78), with high‐risk subjects exhibiting a higher risk of developing CM over time. The discriminative signal captured by the longitudinal time‐to‐event analysis suggests that evaluating risk across the entire follow‐up window captures prognostic echocardiographic information that is not fully reflected by single‐timepoint classification performance. These findings highlight the potential of DL to uncover early imaging markers of CM and support its use in tailoring surveillance intensity and timing for individual patients at elevated risk.

The best performance was observed with the model trained on all available echocardiograms up to 5 years prediagnosis (CM_all_ model), which benefited from the largest sample size and the inclusion of studies closest to CM diagnosis, where structural or functional abnormalities are more likely to be present. This research builds on our group's prior study, which used handcrafted image montages composed of four selected frames from the cardiac cycle to predict future CM using DCNNs [28]. That approach, which achieved an AUROC of 0.64 ± 0.04 and AUPRC of 0.63 ± 0.03, was limited by its reliance on empirically chosen static frames and did not incorporate external validation. In contrast, the present study uses full echocardiographic video clips and a 3D DCNN architecture to capture spatiotemporal features across the cardiac cycle, resulting in improved predictive performance. The use of 3D DCNNs capable of extracting spatiotemporal features was crucial to this approach. Traditional machine learning models that rely on static images or manually engineered features could miss dynamic motion patterns distributed across the cardiac cycle [29]. By standardizing inputs and using histogram matching and temporal subsampling, we preserved relevant physiological information while reducing noise introduced by acquisition variability.

Ground truth CM+/ CM− status was provided by the treating center. To eliminate any potential diagnostic subjectivity and to assess if meaningful predictive signals persist further from CM diagnosis, we trained a second model (CM_1–5_ model) using echocardiograms from only 1 to 5 years prediagnosis. This model excluded echocardiograms within 1 year of CM diagnosis, which are more likely to exhibit overt or borderline changes recognizable on routine surveillance. The performance of the CM_1–5_ model declined in comparison with the CM_all_ model, particularly in terms of AUPRC (0.34 ± 0.11 vs 0.74 ± 0.07). This variation in model performance suggests that the excluded within‐1‐year studies likely carried substantial predictive weight. The decrease in performance can also be attributed to a reduced sample size and increased data imbalance.

Clinically, many childhood cancer survivors are screened with echocardiogram every 2 years [6, 30]. To best inform clinical decision‐making at this timepoint (e.g., follow‐up timing), we trained a third model restricted to the 1‐ to 3‐year prediagnosis period, removing echocardiographic studies further from diagnosis that might not yet reflect early disease changes and are less relevant at this CM screening cadence. Despite the smaller and more imbalanced training dataset, the CM_1–3_ model achieved better AUPRC (0.52 ± 0.15) and F1‐score (0.37 ± 0.19) than the CM_1–5_ model, suggesting that the predictive signal might be stronger in the nearer prediagnostic period, even before overt dysfunction is evident.

Grad‐CAM visualizations suggested that the model's predictions are grounded in clinically plausible cardiac regions. These areas corresponded to structures involved in LV contraction, suggesting that subtle alterations in wall motion, thickening, or texture could inform the model's predictions. Attention patterns were similar between CM+ and CM− studies; we interpret this as an expected property of the task rather than a shortcoming of the model, since the myocardial wall and papillary muscles are the only structures carrying diagnostically relevant information and should draw model attention in both classes. This is consistent with Grad‐CAM's function as a coarse class‐discriminative localization method rather than a pixel‐level feature explanation [27]. Benchmarking against expert‐defined ground truth in other imaging domains has shown that Grad‐CAM, while it localizes better than other common saliency methods, still falls well short of human‐level accuracy, particularly for smaller or morphologically complex findings [31, 32]. Thus, concordant localization across classes does not imply that the method captures the full basis for the model's decision. Although not yet sufficient for clinical interpretability, visualization tools such as Grad‐CAM offer a foundation for hypothesis generation and could guide future exploration of model explainability.

Our findings align with studies in adult cardiology demonstrating the feasibility of DL‐based echocardiographic interpretation for tasks such as mortality prediction and function estimation [33, 34]. Our work further extends this field by focusing on detecting subtle changes consistent with cardiac remodeling that precede overt systolic dysfunction and by applying DL within a pediatric cancer survivorship context where data scarcity and variability pose major barriers. In the context of pediatric cardiology, automated echocardiographic assessment of ventricular function has been applied, with notable efforts such as EchoNet‐Peds [13] and TOMTEC's AutoLV and AutoSTRAIN [35]. These efforts primarily focused on the assessment of LV size, geometry, and function rather than on predicting CM before observable changes in these parameters occur. In contrast, our models attempted to anticipate future dysfunction. Earlier detection of CM might enable the initiation of therapies such as beta blockers and ACE inhibitors, which could improve outcomes and potentially prevent the disease progression before it becomes clinically evident [36, 37].

From a clinical perspective, these models, once further refined to improve generalization, may serve best as decision‐support tools that augment, rather than replace, current surveillance strategies. For example, DL predictions could flag high‐risk studies for closer review or prompt earlier follow‐up in otherwise asymptomatic patients, particularly when these predictions are integrated with clinical data such as anthracycline dose, age at cancer treatment, or genetic risk factors [38, 39]. Future work could include developing composite risk scores that combine DL outputs with traditional risk factors, integrate multimodal inputs (e.g., ECG, clinical variables), and improve temporal modeling with sequential or transformer‐based architectures. Stakeholder engagement (e.g., clinicians, patients, insurers) will be key to informing how to effectively integrate the model into existing clinical workflows, promote usability, inspire trust, and enhance clinical impact.

Despite encouraging findings, several limitations exist. First, model performance declined on external validation, reflecting the challenges of working with a small, imbalanced dataset and of generalizability across institutions with differing imaging protocols, acquisition settings, and population characteristics [16]. Nonetheless, we recently published a separate analysis that featured a human centrally remeasured echocardiographic dataset that included 52 CM+ and 94 CM‐ subjects from approximately a dozen COG sites (partial overlap with this analysis) and found that combining 7 conventionally measured echocardiographic parameters achieved AUC 0.82 for 2‐year CM prediction in the training dataset (85% of sample) and AUC 0.85 in a randomly selected test dataset (remaining 15% of sample) [40]. This suggests that even relatively small datasets may be able to achieve reasonably accurate prediction, but the utility of models based on centrally remeasured echocardiographic data may be limited in routine clinical practice. A DCNN‐based approach, if robust across different institutional settings, may represent a more generalizable future approach.

Median anthracycline exposure among CM+ subjects was also higher in the development cohort than in the external cohort (374 vs. 250 mg/m^2^; Table 1); it is possible our model partly learned dose‐correlated cardiac morphological features that are less prevalent in the lower‐exposure external population, contributing to the observed performance gap. Future work will explore additional enhancements such as synthetic data augmentation, multi‐view echocardiogram modeling, and incorporation of apical views to improve performance, mitigate overfitting, and enhance generalizability [33, 39]. More sophisticated inter‐site harmonization strategies, such as image‐level learned harmonization using cycle‐consistent generative adversarial networks [40] or feature‐level domain‐invariant learning via adversarial training [41], may also offer further gains beyond the histogram matching approach used here. Second, we did not restrict follow‐up among CM− subjects by requiring a fixed disease‐free interval after their last available echocardiogram. Consequently, some subjects labeled CM− may have developed cardiomyopathy beyond the study period. This conservative approach might attenuate model performance estimates but mirrors real‐world surveillance practice where future disease onset is not always observable within a given study window. Lastly, further evaluation is needed to determine clinical utility, specifically whether early identification of high‐risk patients can lead to effective interventions that improve patient outcomes.

## 5 Conclusion

Our findings highlight the potential of DL to identify subtle echocardiographic markers years before cardiomyopathy diagnosis in pediatric cancer survivors. Although external validation demonstrated limited, near‐chance single‐study discrimination, the model identified longitudinal risk differences in the external cohort. Ultimately, such tools may contribute to future risk‐guided cardiomyopathy surveillance, 2allowing earlier interventions in this high‐risk population.

## Funding

Research reported in this publication was supported by: Rally Foundation (Atlanta, GA); Catherine Holmes Wilkins Charitable Foundation; Unravel Pediatric Cancer Foundation; Seattle Foundation Medical Funds Program; Seattle Children's Research Institute; and National Institutes of Health under award numbers: K23HL183696, R01CA211996, R21CA277746, U10CA180886, and UG1CA189955. The content is solely the responsibility of the authors and does not necessarily represent the official views of the National Institutes of Health. No sponsors or funders played any role in study design; data collection and analysis; decision to publish; or preparation of the manuscript.

## Conflicts of Interest

The authors have no conflicts of interest relevant to this article.

## Supporting information




**Supporting File 1**: echo70643‐sup‐0001‐SuppMat.pdf.

**Supporting File 2**: echo70643‐sup‐0002‐SuppMat.pdf.

## Data Availability

The datasets generated and/or analyzed during the current study are not publicly available due to their sensitive nature. Source code will be made publicly available upon project completion.
