# Supplementary material for: Early Prediction of Cardiomyopathy in Pediatric Cancer Survivors Using Echocardiographic Video‐Based Deep Learning
Source: Echocardiography. 2026 Oct 22;43(10):e70643. doi: 10.1111/echo.70643 (PMC13687910; doi:10.1111/echo.70643)
Supplement: Supplementary file 1 [file ECHO-43-e70643-s001.pdf]

Sharma et al. (2026) Early prediction of cardiomyopathy in pediatric cancer survivors using echocardiographic video-based deep learning: TRIPOD+AI Reporting Checklist

Editable working form | Adapted from Publicly Available Version (January 11th, 2024)

Section / topic	Item	D / E	Checklist item	Reported in section / brief note
**TITLE**<br>Title	**1**	D;E	Identify the study as developing or evaluating the performance of a multivariable prediction model, the target population, and the outcome to be predicted.	Title: "Early prediction of cardiomyopathy in pediatric cancer survivors using echocardiographic video-based deep learning."
**ABSTRACT**<br>Abstract	**2**	D;E	See TRIPOD+AI for Abstracts checklist.	N/A: Not a conference abstract
**INTRODUCTION**<br>Background	**3a**	D;E	Explain the healthcare context (including whether diagnostic or prognostic) and rationale for developing or evaluating the prediction model, including references to existing models.	Introduction: Clinical context and rationale described. Companion conventional measurement model discussed.
**INTRODUCTION**<br>Background	**3b**	D;E	Describe the target population and the intended purpose of the prediction model in the context of the care pathway, including its intended users (e.g., healthcare professionals, patients, public).	Introduction describes the target population and precision-guided surveillance purpose. The intended future use would be as clinician-facing decision support during routine echocardiographic surveillance, potentially identifying studies that warrant closer review or earlier follow-up. The present proof-of-concept model is not intended for clinical deployment or independent treatment decisions.
**INTRODUCTION**<br>Background	**3c**	D;E	Describe any known health inequalities between sociodemographic groups.	Discussed; Incomplete race and ethnicity data limited our ability to assess potential healthcare inequalities or differential model performance across sociodemographic groups.
**INTRODUCTION**<br>Objectives	**4**	D;E	Specify the study objectives, including whether the study describes the development or validation of a prediction model (or both).	Introduction states that the study developed DL models for future CM prediction across multiple prediagnostic periods. We developed and internally evaluated 3D DCNN models for predicting future CM and independently evaluated the best-performing model in two external institutional cohorts.
**METHODS**<br>Data	**5a**	D;E	Describe the sources of data separately for the development and evaluation datasets (e.g., randomised trial, cohort, routine care or registry data), the rationale for using these data, and representativeness of the data.	Study population section describes the retrospective data source, use of Center 1 for development/internal validation, and Centers 2–3 for external validation. Cohorts were assembled from available eligible survivors with digitally archived PSAX videos and were not population-based samples.
**METHODS**<br>Data	**5b**	D;E	Specify the dates of the collected participant data, including start and end of participant accrual; and, if applicable, end of follow-up.	The echocardiogram dates ranged from 2004 to 2023. Median echocardiogram year and IQR by cohort and case status are shown in Table 1.
**METHODS**<br>Participants	**6a**	D;E	Specify key elements of the study setting (e.g., primary care, secondary care, general population) including the number and location of centres.	Study population section identifies three participating large children's hospitals and their respective development and external-validation roles.
**METHODS**<br>Participants	**6b**	D;E	Describe the eligibility criteria for study participants.	Study population section reports age, cancer history, cardiotoxic treatment exposure, post-treatment survival requirement, CM+/CM− definitions, and exclusion criteria.
**METHODS**<br>Participants	**6c**	D;E	Give details of any treatments received, and how they were handled during model development or evaluation, if relevant.	Relevant anthracycline and chest-radiotherapy exposures are described and reported in Table 1. Cancer-treatment exposures were used to characterize the cohorts but were not provided as inputs to the DCNN.
**METHODS**<br>Data preparation	**7**	D;E	Describe any data pre-processing and quality checking, including whether this	Echocardiographic data selection and processing section describes view selection, quality

Section / topic	Item	D / E	Checklist item	Reported in section / brief note
			was similar across relevant sociodemographic groups.	review, random clip selection, masking, frame-rate normalization, temporal subsampling, resizing, histogram matching, grayscale conversion, and tested data augmentation. The same view-selection and preprocessing pipeline were applied across all cohorts and did not use sociodemographic or clinical characteristics.
**METHODS** Outcome	**8a**	D;E	Clearly define the outcome that is being predicted and the time horizon, including how and when assessed, the rationale for choosing this outcome, and whether the method of outcome assessment is consistent across sociodemographic groups.	Study population and Data labeling sections define CM, explain its clinical rationale, and specify the CMall, CM1–5, and CM1–3 prediagnostic periods. The same CM definition was applied across centers; the exact interval from each CMall echocardiogram to diagnosis varied within the five-year window.
**METHODS** Outcome	**8b**	D;E	If outcome assessment requires subjective interpretation, describe the qualifications and demographic characteristics of the outcome assessors.	Clinical EF/SF values and treatment records were extracted by trained research personnel.
**METHODS** Outcome	**8c**	D;E	Report any actions to blind assessment of the outcome to be predicted.	CM status was established from clinical echocardiographic reports and treatment records before model development and was therefore determined without knowledge of DCNN predictions. Echocardiograms were not centrally remeasured for this analysis.
**METHODS** Predictors	**9a**	D	Describe the choice of initial predictors (e.g., literature, previous models, all available predictors) and any pre-selection of predictors before model building.	DCNN input consisted of processed PSAX echocardiographic videos. No clinical variables, treatment exposures, or conventional echocardiographic measurements were included.
**METHODS** Predictors	**9b**	D;E	Clearly define all predictors, including how and when they were measured (and any actions to blind assessment of predictors for the outcome and other predictors).	Echocardiographic data selection and processing section defines the PSAX input, clip selection, 16-frame sampling, frame rate, pixel dimensions, masking, and image normalization.
**METHODS** Predictors	**9c**	D;E	If predictor measurement requires subjective interpretation, describe the qualifications and demographic characteristics of the predictor assessors.	PSAX clips were identified by trained sonographers or study physicians.
**METHODS** Sample size	**10**	D;E	Explain how the study size was arrived at (separately for development and evaluation), and justify that the study size was sufficient to answer the research question. Include details of any sample size calculation.	Sample size was determined by the number of eligible CM cases and controls with available digitized echocardiographic videos. No prospective statistical sample-size calculation was performed because this was a retrospective proof-of-concept study.
**METHODS** Missing data	**11**	D;E	Describe how missing data were handled. Provide reasons for omitting any data.	The DCNN did not use tabular clinical variables, so no statistical imputation was required for model inputs.
**METHODS** Analytical methods	**12a**	D	Describe how the data were used (e.g., for development and evaluation of model performance) in the analysis, including whether the data were partitioned, considering any sample size requirements.	DCNN architecture and performance section describes the patient-level 60:20:20 development split, fivefold procedure, fixed internal hold-out set, and external evaluation.
**METHODS** Analytical methods	**12b**	D	Depending on the type of model, describe how predictors were handled in the analyses (functional form, rescaling, transformation, or any standardisation).	Predictor processing included frame-rate normalization, temporal subsampling, spatial resizing, histogram matching, grayscale conversion, and standardized 16-frame input dimensions.
**METHODS** Analytical methods	**12c**	D	Specify the type of model, rationale, all model-building steps, including any hyperparameter tuning, and method for internal validation.	Methods and Table S1 report the 3D DCNN architecture, optimizer, loss function, regularization, dropout, tuning, internal-validation procedure, and hyperparameter tuning.
**METHODS** Analytical methods	**12d**	D;E	Describe if and how any heterogeneity in estimates of model parameter values and model performance was handled and quantified across clusters (e.g., hospitals, countries).	External performance was estimated separately for Centers 2 and 3 to describe cross-center heterogeneity. Heterogeneity in estimates across clusters was handled appropriately for performance and time-to-event analyses, as described.
**METHODS** Analytical methods	**12e**	D;E	Specify all measures and plots used (and their rationale) to evaluate model performance (e.g., discrimination, calibration, clinical utility) and, if relevant,	Methods specify AUROC, AUPRC, sensitivity, specificity, PPV, NPV, F1-score, accuracy, Cox regression, hazard ratio, and log-rank testing. Calibration plots for all three cohorts

Section / topic	Item	D / E	Checklist item	Reported in section / brief note
			to compare multiple models.	(development + two external holdout) are included.
**METHODS** Analytical methods	**12f**	E	Describe any model updating (e.g., recalibration) arising from the model evaluation, either overall or for particular sociodemographic groups or settings.	N/A: No model updating or recalibration was performed after external evaluation. Site-specific thresholds were exploratory and did not update the trained model.
**METHODS** Analytical methods	**12g**	E	For model evaluation, describe how the model predictions were calculated (e.g., formula, code, object, application programming interface).	External predictions were generated by applying the five fold-specific CMall models without retraining.
**METHODS** Class imbalance	**13**	D;E	If class imbalance methods were used, state why and how this was done, and any subsequent methods to recalibrate the model or the model predictions.	AUPRC was used because of outcome imbalance. No additional imbalance methods were used.
**METHODS** Fairness	**14**	D;E	Describe any approaches that were used to address model fairness and their rationale.	N/A: No fairness-specific modeling or mitigation approach was used.
**METHODS** Model output	**15**	D	Specify the output of the prediction model (e.g., probabilities, classification). Provide details and rationale for any classification and how the thresholds were identified.	Model output is a Softmax CM+ probability, and classification used a Youden-index threshold determined exclusively from Center 1 data. Site-specific thresholds were examined only as an exploratory analysis.
**METHODS** Training versus evaluation	**16**	D;E	Identify any differences between the development and evaluation data in healthcare setting, eligibility criteria, outcome, and predictors.	The same eligibility criteria, CM definition, video input, and preprocessing pipeline were used across cohorts. Imaging protocols, acquisition settings, population characteristics, demographic-data completeness, and anthracycline exposures differed across centers.
**METHODS** Ethical approval	**17**	D;E	Name the institutional research board or ethics committee that approved the study and describe the participant-informed consent or the ethics committee waiver of informed consent.	Ethics in research section identifies the Fred Hutchinson Cancer Center IRB and NIH Central IRB, provides protocol numbers, and describes the waiver of informed consent.
**OPEN SCIENCE** Funding	**18a**	D;E	Give the source of funding and the role of the funders for the present study.	Funding section identifies all funding sources and states that funders had no role in study design, data collection or analysis, publication decisions, or manuscript preparation.
**OPEN SCIENCE** Conflicts of interest	**18b**	D;E	Declare any conflicts of interest and financial disclosures for all authors.	Conflict-of-interest section declares all potentially relevant conflicts.
**OPEN SCIENCE** Protocol	**18c**	D;E	Indicate where the study protocol can be accessed or state that a protocol was not prepared.	Add: "A detailed study protocol and statistical analysis plan were not deposited in a public repository before analysis."
**OPEN SCIENCE** Registration	**18d**	D;E	Provide registration information for the study, including register name and registration number, or state that the study was not registered.	N/A: This retrospective model-development and evaluation study was not prospectively registered.
**OPEN SCIENCE** Data sharing	**18e**	D;E	Provide details of the availability of the study data.	A detailed note regarding Data and Code Availability is included.
**OPEN SCIENCE** Code sharing	**18f**	D;E	Provide details of the availability of the analytical code.	A detailed note regarding Data and Code Availability is included.
**PATIENT & PUBLIC INVOLVEMENT** Patient & Public Involvement	**19**	D;E	Provide details of any patient and public involvement during the design, conduct, reporting, interpretation, or dissemination of the study or state no involvement.	N/A: Patients and members of the public were not directly involved in the design, conduct, interpretation, reporting, or dissemination planning of this retrospective study.
**RESULTS** Participants	**20a**	D;E	Describe the flow of participants through the study, including the number of participants with and without the outcome and, if applicable, a summary of the follow-up time. A diagram may be helpful.	Results report 74 development-cohort and 153 external-cohort patients, CM+/CM− counts, study counts, and median follow-up.

Section / topic	Item	D / E	Checklist item	Reported in section / brief note
**RESULTS** Participants	**20b**	D;E	Report the characteristics overall and, where applicable, for each data source or setting, including the key dates, key predictors (including demographics), treatments received, sample size, number of outcome events, follow-up time, and amount of missing data. A table may be helpful. Report any differences across key demographic groups.	Results and Table 1 report demographics, cancer type, treatment exposure, sample size, outcome counts, diagnosis periods, and follow-up.
**RESULTS** Participants	**20c**	E	For model evaluation, show a comparison with the development data of the distribution of important predictors (demographics, predictors, and outcome).	Table 1 compares development and external-validation cohorts with respect to demographic characteristics, cancer characteristics, treatment exposures, outcome frequency, and follow-up. External centers are presented as a pooled cohort.
**RESULTS** Model development	**21**	D;E	Specify the number of participants and outcome events in each analysis (e.g., for model development, hyperparameter tuning, model evaluation).	Results report total patients and studies in the development and external cohorts.
**RESULTS** Model specification	**22**	D	Provide details of the full prediction model (e.g., formula, code, object, application programming interface) to allow predictions in new individuals and to enable third-party evaluation and implementation, including any restrictions to access or re-use (e.g., freely available, proprietary).	Figure S1 illustrates the model architecture, Table S1 provides final hyperparameters, and Methods describe the main layers and training configuration.
**RESULTS** Model performance	**23a**	D;E	Report model performance estimates with confidence intervals, including for any key subgroups (e.g., sociodemographic). Consider plots to aid presentation.	Tables 2–3 report discrimination and classification performance as mean ± SD; Tables S2–S3 report Wilson 95% CIs for sensitivity and specificity; the Cox model reports an HR with 95% CI. Calibration plots are also included for all three cohorts.
**RESULTS** Model performance	**23b**	D;E	If examined, report results of any heterogeneity in model performance across clusters.	Model performance is reported separately for Centers 2 and 3. No formal heterogeneity test was performed because only two external centers were available.
**RESULTS** Model updating	**24**	E	Report the results from any model updating, including the updated model and subsequent performance.	N/A: No model updating or recalibration was performed. Exploratory site-specific thresholds did not modify the trained model.
**DISCUSSION** Interpretation	**25**	D;E	Give an overall interpretation of the main results, including issues of fairness in the context of the objectives and previous studies.	Discussion interprets internal and external performance in relation to the study objectives and prior DL studies.
**DISCUSSION** Limitations	**26**	D;E	Discuss any limitations of the study (such as a non-representative sample, sample size, overfitting, missing data) and their effects on any biases, statistical uncertainty, and generalizability.	Limitations are explicitly stated, includeing small and imbalanced datasets, reduced external performance, interinstitutional differences, anthracycline-dose imbalance, and possible outcome misclassification.
**DISCUSSION** Usability in current care	**27a**	D	Describe how poor quality or unavailable input data (e.g., predictor values) should be assessed and handled when implementing the prediction model.	Suboptimal-quality studies were retained to represent clinical variability.
**DISCUSSION** Usability in current care	**27b**	D	Specify whether users will be required to interact in the handling of the input data or use of the model, and what level of expertise is required of users.	The current pipeline requires trained sonographers or study physicians to identify appropriate PSAX clips.
**DISCUSSION** Usability in current care	**27c**	D;E	Discuss any next steps for future research, with a specific view to applicability and generalizability of the model.	Discussion identifies future work involving multiview modeling, harmonization, multimodal inputs, synthetic augmentation, temporal modeling, and stakeholder engagement.
