# Supplementary material for: Early Prediction of Cardiomyopathy in Pediatric Cancer Survivors Using Echocardiographic Video‐Based Deep Learning
Source: Echocardiography. 2026 Oct 22;43(10):e70643. doi: 10.1111/echo.70643 (PMC13687910; doi:10.1111/echo.70643)
Supplement: Supplementary file 2 [file ECHO-43-e70643-s002.pdf]

Supplemental Appendix

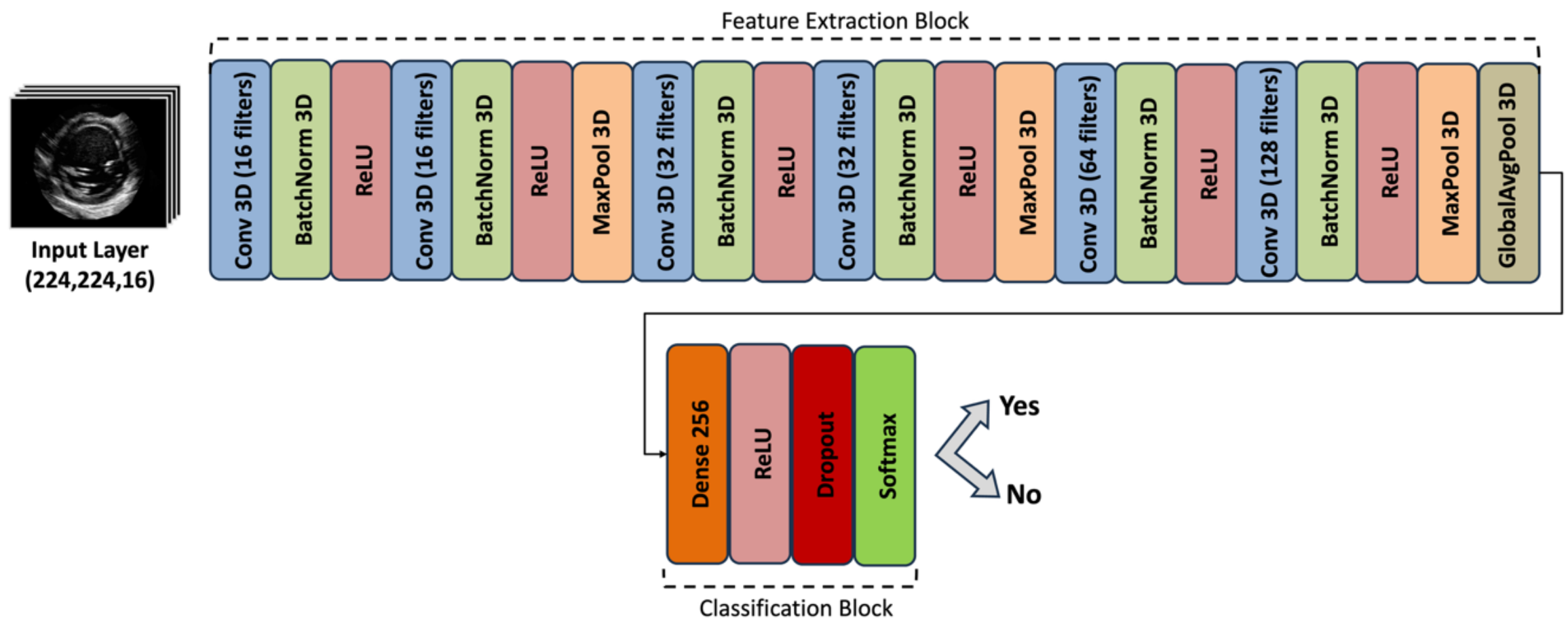

Figure S1: 3D CNN architecture: The architecture is composed of two main parts: a feature extraction block and a classification block. The feature extraction component applies a series of 3D convolutional layers, each followed by batch normalization, ReLU activation, and 3D max pooling operations. A global average pooling layer is used at the end to condense the spatial information into a compact feature representation. This output is passed to the classification block, which includes a fully connected layer with ReLU activation, a dropout layer to reduce overfitting and a SoftMax function that produces class probabilities indicating the presence or absence of cardiomyopathy.

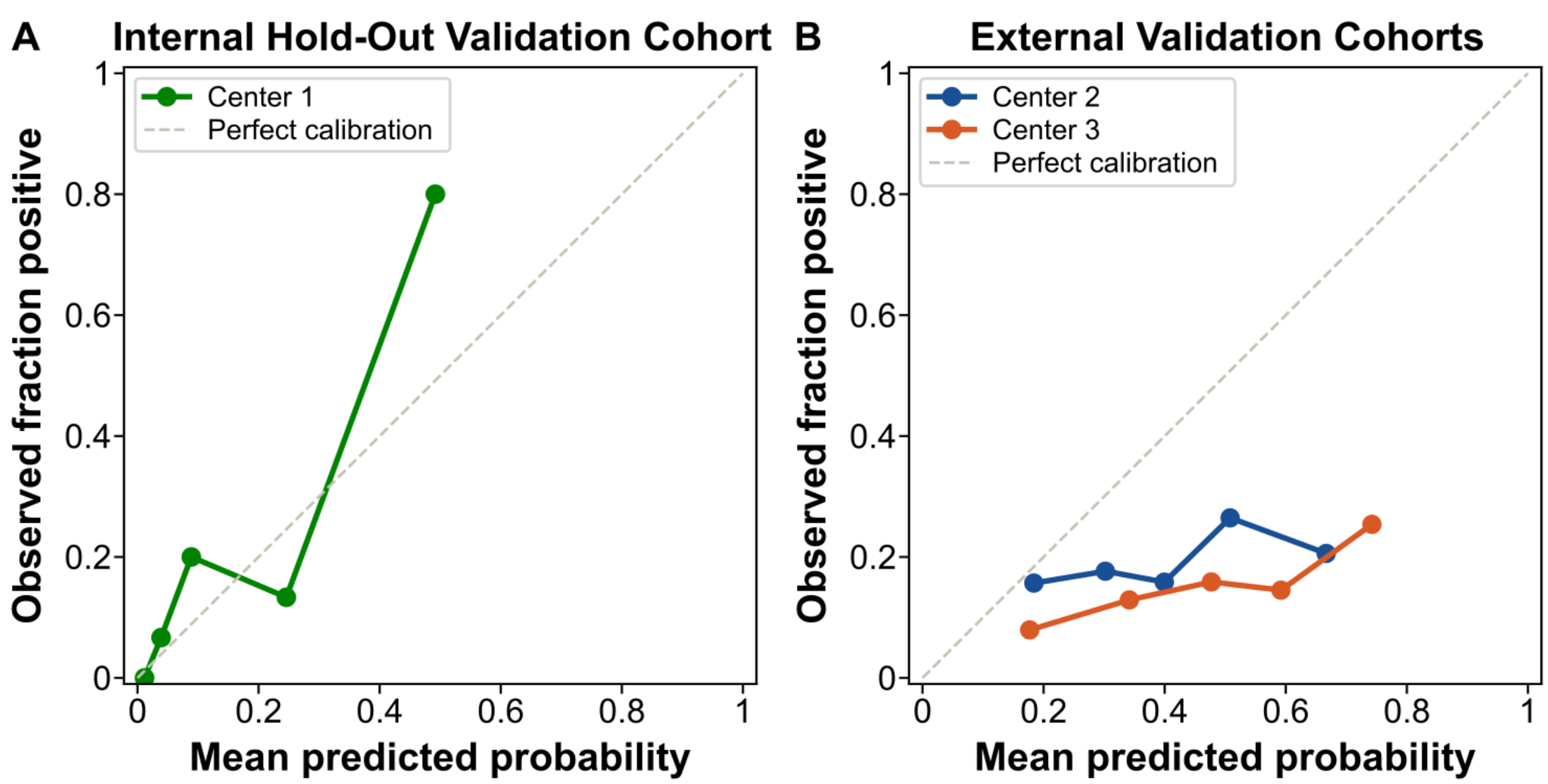

Figure S2: Probability calibration plots for the internal hold-out cohort (A) and the external validation cohorts.

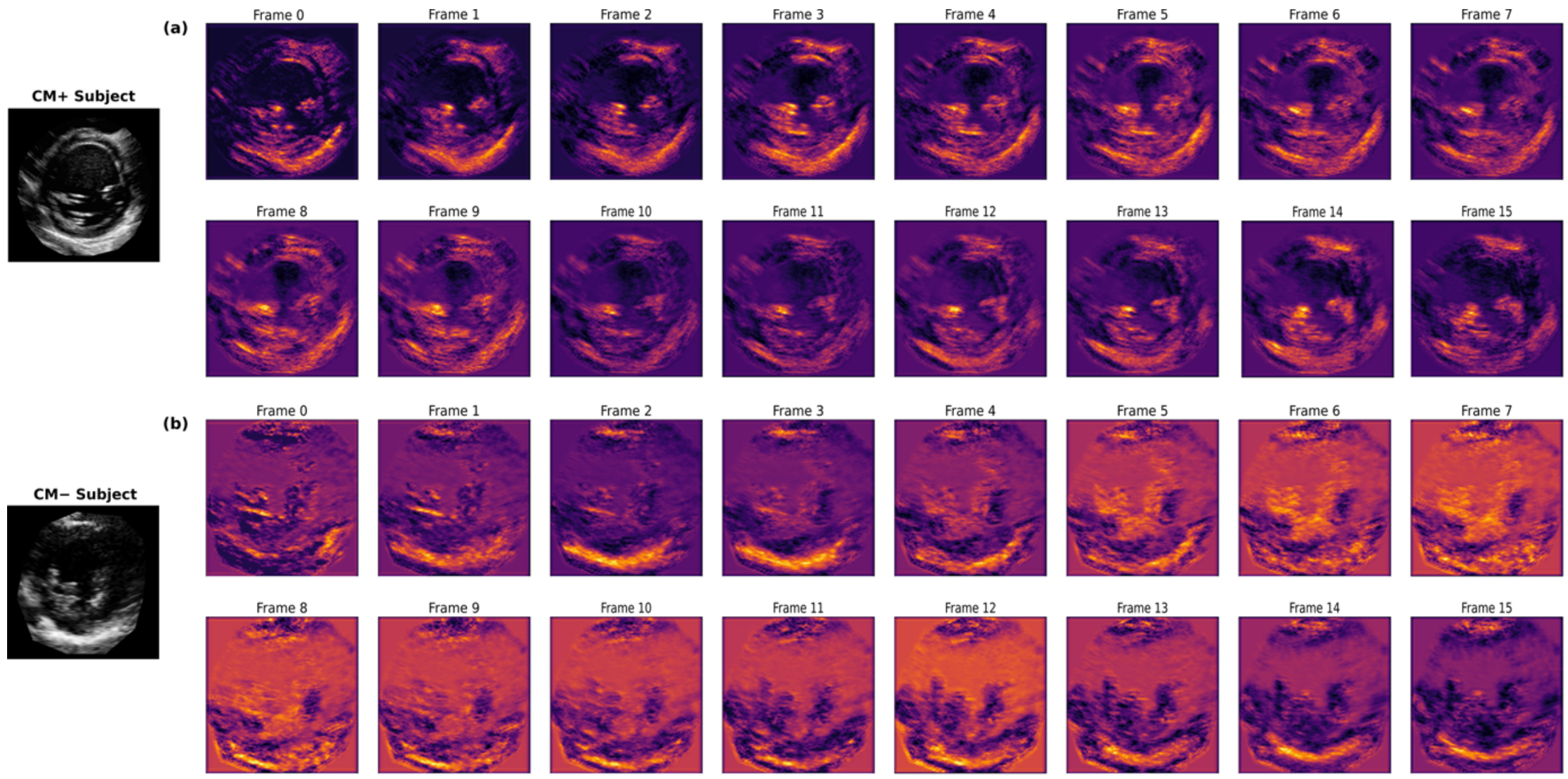

Figure S3: Full-frame Grad-CAM heatmaps for the CM+ and CM– examples shown in Figure 3. Each of the 16 input frames is displayed with overlaid Grad-CAM maps, providing a complete temporal view of model attention across the cardiac cycle. Brighter regions represent features with greater influence on the model's prediction.

Table S1: Hyperparameters for 3D CNN model

**Number of epochs**	100
**Batch size**	32
**Learning rate**	0.00001
**Dropout rate**	0.3
**Optimizer**	Adam's optimizer

Table S2: Sensitivity and specificity metrics for the two external holdout cohorts, including 95% Wilson score confidence intervals. These were computed on the ensembled predictions across all five cross-validation folds.

Metrics	Center 2	Center 3
**Sensitivity (95% CI)**	0.60 (0.50–0.69)	0.66 (0.53–0.78)
**Specificity (95% CI)**	0.55 (0.50–0.60)	0.53 (0.47–0.59)

Table S3: Sensitivity and specificity metrics for the two external holdout cohorts, repeated with site-specific operating thresholds (Youden index applied independently within Center 2 and Center 3); 95% Wilson score confidence intervals are included. These were computed on the ensembled predictions across all five cross-validation folds.

Metrics	Center 2	Center 3
**Sensitivity (95% CI)**	0.67 (0.60–0.74)	0.75 (0.70–0.80)
**Specificity (95% CI)**	0.53 (0.48–0.58)	0.47 (0.43–0.50)
